# A comprehensive re-evaluation of the psychometric properties of the Kaufman survey of early academic and language skills in Turkish children aged 48–72 months: multidimensional structure, measurement invariance, and validity evidence

**DOI:** 10.3389/fpsyg.2026.1871332

**Published:** 2026-07-27

**Authors:** Elif Çağlak, Adalet Kandır

**Affiliations:** Department of Early Childhood Education, Faculty of Education, Gazi University, Ankara, Türkiye

**Keywords:** cross-cultural assessment, early academic skills, early childhood, K-SEALS, language development, measurement invariance, psychometrics

## Abstract

**Background:**

The assessment of early academic and language skills across different cultural contexts requires robust psychometric validation. Although the Kaufman Survey of Early Academic and Language Skills (K-SEALS) has been adapted into Turkish, there is a need to re-evaluate its psychometric properties using contemporary analytical approaches.

**Methods:**

This study aimed to examine the psychometric properties of the Turkish version of K-SEALS in a sample of 385 children aged 48–72 months attending preschool institutions in Konya, Turkey. Confirmatory factor analysis (CFA) using Diagonally Weighted Least Squares (DWLS) estimation was conducted to test the multidimensional structure of the scale. Measurement invariance across gender was examined using multi-group confirmatory factor analysis, whereas developmental sensitivity was evaluated by examining the relationships between chronological age and subtest scores. Convergent and discriminant validity analyses were also performed. Reliability was assessed using internal consistency and test–retest methods.

**Results:**

The findings supported the theoretically proposed multidimensional structure of the scale, with model fit indices within acceptable ranges. Measurement invariance across gender was established, indicating structural equivalence. Convergent and discriminant validity analyses confirmed that the K-SEALS dimensions represent related but distinct constructs. Reliability results demonstrated strong internal consistency and temporal stability. High inter-factor correlations suggested that early academic and language skills function as an integrated developmental system.

**Conclusion:**

The results provide further psychometric evidence for the validity and reliability of the Turkish version of K-SEALS. The findings provide additional evidence for the validity and reliability of the Turkish version of K-SEALS.

## 1 Introduction

Early childhood is a critical developmental period in which the foundations of cognitive, linguistic, and academic development are established. The skills acquired during this period not only affect school readiness but also directly influence academic achievement in later educational life. Current research indicates that early academic and language skills are strong predictors of both short-term school adjustment and long-term academic trajectories (Bailey et al., 2020; Duncan et al., 2007; Duncan et al., 2022).

Today, rapidly changing social structures, technological developments, and the transformation of learning environments have significantly altered the nature of cognitive and linguistic stimuli encountered by children at an early age. In particular, increased access to digital content and the diversification of learning experiences may lead to differences in children’s linguistic and cognitive processing (Hirsh-Pasek et al., 2015; Plowman et al., 2012). This transformation highlights the need to view early childhood assessment instruments as context-sensitive measures that require periodic re-evaluation rather than as static tools.

Early academic and language skills are among the fundamental indicators for understanding children’s learning processes, identifying individual differences, and planning educational interventions. These skills constitute a core component of school readiness and are particularly associated with early literacy, vocabulary knowledge, and basic mathematical concepts (Claessens et al., 2009; Snow and Matthews, 2016). However, the development of these skills is not limited solely to cognitive processes; executive functions (attention control, working memory, and cognitive flexibility) are among the important determinants of early academic performance (Blair and Raver, 2015; Cirino, 2023; McClelland and Cameron, 2011). This multidimensional nature suggests that assessment tools focusing solely on overall achievement may not adequately capture the complexity of early academic and language development. In particular, findings suggesting that mathematical development depends not only on numerical skills but also on mathematical language skills (Purpura and Logan, 2015) clearly demonstrate the intertwined nature of language and academic skills.

Assessment instruments developed to evaluate early academic skills enable the systematic examination of children’s early literacy, vocabulary knowledge, and foundational academic competencies (Lonigan et al., 2011). However, the psychometric strength of these tools is a critical requirement for the reliability of the results obtained. Validity and reliability evidence for assessment instrument are not limited to one-time analyses, but rather constitute dynamic structures that must be continuously re-tested across different samples, time periods, and contexts (DeVellis, 2017; Kline, 2016).

One of the measurement instruments developed in this context is the Kaufman Survey of Early Academic and Language Skills (K-SEALS). K-SEALS aims to evaluate children’s early academic and language skills in a multidimensional manner and is used as an instrument with the potential to predict early academic development (Kaufman and Kaufman, 1993; Taylor et al., 2013). K-SEALS addresses academic and language skills in an integrated manner, which is theoretically consistent with the holistic nature of early childhood development. Furthermore, the Cattell–Horn–Carroll (CHC) theory, which explains the relationship between academic skills and cognitive abilities, emphasizes that academic performance emerges as a result of the interaction of different cognitive components (McGrew and Wendling, 2010). This theoretical framework indicates that measurement instruments should not only reflect observed performance but also indirectly represent the underlying cognitive structures.

K-SEALS has been widely used in the international literature not only in psychometric studies but also in research examining the relationships between early academic and language skills and various developmental and educational variables. In studies conducted in Turkey, the scale has been examined in relation to self-regulation, social adaptation, and early academic skills, and it has been revealed that these skills show strong relationships with each other (Uyanık et al., 2017; Gözüm and Uyanık Aktulun, 2021). These findings suggest that K-SEALS is positioned not only as a measurement instrument but also as a fundamental construct indicator within multivariate models explaining early childhood development.

However, although measurement instruments provide validity and reliability evidence at the time they are developed, these properties are not fixed and unchanging. Psychometric properties may be reshaped depending on sample characteristics, application context, and the statistical techniques used. Therefore, re-examining measurement instruments across different samples and in line with developing analytical techniques is important for strengthening validity and reliability evidence (Embretson and Reise, 2000).

The Turkish adaptation study conducted by Uyanık and Kandır (2014) primarily focused on establishing linguistic equivalence and providing initial evidence for validity and reliability. However, advances in psychometric methodology now allow more comprehensive evaluations, including multidimensional confirmatory factor analysis, measurement invariance testing, convergent and discriminant validity, and detailed item-level analyses. Accordingly, the present study extends the available psychometric evidence by applying these contemporary analytical approaches to the Turkish version of K-SEALS. However, there are significant differences between the period in which this adaptation study was conducted and today’s learning contexts. In particular, digitalization, the transformation of learning environments, and the diversification of early experiences may have influenced new variables that may affect children’s measured cognitive and linguistic performance. In addition, the widespread use of advanced statistical approaches in recent years such as confirmatory factor analysis, measurement invariance, and categorical data models has made the re-evaluation of existing measurement instruments not only recommended but methodologically necessary.

Despite previous adaptation studies, an important opportunity remains to further strengthen the psychometric evidence for the Turkish version of K-SEALS. Since the original adaptation study, advances in psychometric methodology have enabled more comprehensive evaluations of measurement instruments. In particular, contemporary approaches such as multidimensional confirmatory factor analysis, measurement invariance testing, and item-level analyses provide additional evidence regarding the validity and reliability of assessment instruments. Therefore, the present study re-evaluates the psychometric properties of the Turkish version of K-SEALS using these contemporary analytical approaches and examines its multidimensional structure, measurement invariance, and validity and reliability evidence within a comprehensive framework. Rather than replicating the previous adaptation study, the present study builds upon and extends the existing psychometric evidence by applying current methodological standards.

### 1.1 Purpose of the study and research questions

The aim of this study was to comprehensively evaluate the psychometric properties of the Turkish version of the Kaufman Survey of Early Academic and Language Skills (K-SEALS) in a sample of Turkish children aged 48–72 months. Specifically, the study examined the instrument using contemporary psychometric approaches while interpreting the findings within their cultural and linguistic context.

The following research questions were addressed:

1. Is the theoretical factor structure of the K-SEALS supported according to the results of confirmatory factor analysis (CFA)?
2. Do the K-SEALS subtests and scales demonstrate adequate internal consistency and test–retest reliability?
3. Does the scale provide sufficient evidence for convergent and discriminant validity?
4. Are the relationships among the K-SEALS dimensions consistent with the proposed theoretical structure?
5. Does item-level psychometric performance support the adequacy of the measurement model?
6. Does the scale demonstrate evidence of developmental sensitivity across age?
7. Does the scale demonstrate measurement invariance across gender groups?
8. Are age-related differences in K-SEALS scores consistent with developmental expectations?

## 2 Materials and methods

### 2.1 Research design

This study employed a quantitative methodological design to evaluate the psychometric properties of the Turkish version of the Kaufman Survey of Early Academic and Language Skills (K-SEALS). The primary aim of the study was to re-evaluate the construct validity, measurement invariance, and reliability characteristics of K-SEALS in a Turkish sample by using contemporary psychometric approaches and analyses based on categorical data models.

Although the Turkish adaptation of K-SEALS has been previously conducted, re-examining the psychometric properties of measurement instruments across different samples, age groups, and data structures is considered essential for evaluating the validity and generalizability of previous findings (Brown, 2015; Kline, 2016). This approach is consistent with contemporary measurement theories, which emphasize that validity is not a one-time property but a cumulative structure that must be continuously tested across different contexts.

Accordingly, the study was designed as a cross-sectional methodological investigation aimed at confirming the existing factor structure of the instrument. Because the primary objective was to confirm the previously proposed theoretical structure in a new sample, confirmatory rather than exploratory analytical procedures were employed. Within this framework, confirmatory factor analysis (CFA), measurement invariance analysis, and reliability analyses were conducted to examine the psychometric properties of the Turkish version of K-SEALS.

Considering the dichotomous nature of the items, the Diagonally Weighted Least Squares (DWLS) estimation method was employed because it provides robust parameter estimates when the assumption of multivariate normality is violated (Brown, 2015).

In the study, confirmatory factor analysis was used to evaluate the construct validity of the measurement instrument; measurement invariance analyses were conducted to determine measurement equivalence across different groups; and internal consistency and test–retest analyses were used to determine the reliability of the scale. Measurement invariance was evaluated sequentially at the configural, metric, and scalar levels to provide evidence regarding the comparability of the instrument across gender groups.

Collectively, these analyses provided a comprehensive evaluation of the structural validity and reliability of the Turkish version of K-SEALS.

### 2.2 Participants

The study group of this research consists of a total of 385 children aged 48–72 months who were attending public or private preschool education institutions affiliated with the Turkish Ministry of National Education in the central districts of Konya during the 2025–2026 academic year. This age range was preferred because it represents a critical developmental period during which early academic and language skills develop rapidly and individual differences become more evident. Following approval from the Konya Provincial Directorate of National Education, public and private preschool institutions located in the central districts of Konya were invited to participate in the study. Children were subsequently recruited from the institutions that agreed to participate from these institutions after written informed parental consent had been obtained.

In the selection of the study group, criterion sampling, one of the purposive sampling methods, was used. Accordingly, the inclusion criteria were as follows:

- The child being between 48 and 72 months of age
- The child attending a preschool education institution
- The absence of any diagnosed developmental disability
- Obtaining voluntary parental consent

These criteria ensured that the data were collected from a group of typically developing children consistent with the target population of the measurement instrument and contributed to increasing the internal validity of the psychometric analyses. The sample size was evaluated based on the recommended sample size criteria for confirmatory factor analysis and was considered sufficient when taking into account model complexity and the number of parameters (Kline, 2016).

Of the participants, 200 were female (51.9%) and 185 were male (48.1%), indicating that the sample had a balanced distribution in terms of gender. Regarding age, 93 children (24.2%) were 4 years old, 133 (34.5%) were 5 years old, 130 (33.8%) were 6 years old, and 29 (7.5%) were 7 years old. Thus, approximately two-thirds of the sample consisted of children aged 5–6 years. When the socio-demographic characteristics of the sample were examined, it was observed that the educational level of the parents was predominantly at the undergraduate level or higher. This characteristic should be considered when interpreting the findings and may represent a limitation with respect to the generalizability of the results.

In addition, it was observed that the majority of the children were attending kindergarten, while a smaller proportion attended preschool classes, and that the distribution of the number of siblings was relatively balanced. This distribution indicates that early learning experiences occurred within a relatively homogeneous educational context. Overall, the sample largely consisted of preschool children aged 5–6 years, most of whom had access to institutional early childhood education and whose parents had relatively high levels of education. These characteristics provide a contextual framework for interpreting the psychometric findings and should be carefully considered, particularly in terms of cross-cultural comparisons.

### 2.3 Instruments

Data were collected using the Personal Information Form and the Kaufman Survey of Early Academic and Language Skills (K-SEALS).

Personal Information Form: The Personal Information Form was developed by the researcher to obtain descriptive data about the children and their parents included in the study. The form includes variables such as gender, age, number of siblings, birth order, duration of preschool attendance, parents’ educational level, occupations, and ages. These variables were used to characterize the socio-demographic profile of the sample and to facilitate the contextual interpretation of the psychometric findings.

Kaufman Survey of Early Academic and Language Skills (K-SEALS): In this study, the Turkish version of the Kaufman Survey of Early Academic and Language Skills (K-SEALS), developed by Kaufman and Kaufman (1993), was used. K-SEALS is a standardized measurement instrument developed to assess early academic and language skills of children aged 36–83 months.

The scale consists of three main subtests:

1. Vocabulary
2. Numbers, Letters and Words
3. Articulation Skills

Through these subtests, children’s expressive and receptive language skills, early academic performance, and knowledge of symbolic systems are assessed. This structure is consistent with theoretical approaches, including CHC theory, which emphasize that early academic development emerges from the interaction of linguistic and cognitive components.

When the psychometric findings of the original version of K-SEALS are examined, it is reported that the scale has high internal consistency coefficients (generally ranging between 0.80–0.90) and strong test–retest reliability values (Kaufman and Kaufman, 1993). In addition, it is stated that the scale shows significant relationships with various achievement and intelligence tests and validly measures early academic and language skills. Accordingly, K-SEALS has been widely used in international research on school readiness, early academic development, and educational interventions.

The Turkish adaptation of K-SEALS was conducted by Uyanık and Kandır (2014), and validity and reliability evidence for the Turkish version of the scale was established. During the adaptation process, linguistic equivalence was ensured, and it was determined that the factor structure of the scale was largely preserved. The psychometric findings described above refer to the Turkish adaptation study conducted by Uyanık and Kandır (2014), which established the initial validity and reliability evidence for the Turkish version of K-SEALS. The present study builds upon this foundation by re-evaluating the psychometric properties of the instrument in an independent sample using contemporary psychometric approaches.

In this study, the Turkish version of K-SEALS was taken as a basis, and the psychometric findings obtained in previous studies were accepted as a reference. However, considering that the psychometric properties of measurement instruments may vary depending on time, sample, and analytical techniques (DeVellis, 2017; Kline, 2016), the scale was re-evaluated using contemporary analytical methods.

In the present study, data were collected using the original 100-item version of K-SEALS. Because several psychometric models (subtests, skill scales, and the composite structure) were evaluated separately, item retention decisions were made independently for each measurement model. Consequently, an item removed from a specific subtest model because of inadequate psychometric performance was not necessarily removed from other models if it contributed to theoretical integrity and acceptable overall model fit. Therefore, the number of retained items varied depending on the measurement model being evaluated.

During the item removal process, not only statistical criteria but also theoretical meaningfulness and content integrity were taken into account; thus, a structure that preserves the structural validity of the measurement instrument while improving model fit was obtained. This approach provides a comprehensive psychometric evaluation that extends previous adaptation findings by examining the item-level psychometric functioning of the scale using contemporary analytical methods.

### 2.4 Ethical considerations

This study was conducted in accordance with relevant ethical principles and standards. In order to carry out the research, ethical approval was obtained from the Ethics Committee of Gazi University, and the study process was conducted within the framework of official permissions obtained from the Turkish Ministry of National Education.

For all children participating in the study, informed voluntary consent was obtained from their parents, and participation in the study was entirely based on voluntariness. The identities of the participants were kept confidential, the obtained data were anonymized, and they were used solely for scientific purposes.

During the research process, data collection practices were conducted by considering the developmental characteristics of children and in accordance with ethical sensitivity and principles of psychological well-being. Necessary precautions were taken to ensure that children were not exposed to any physical or emotional risks during the implementation, and the process was carried out using a child-centered approach.

In this study, the necessary permissions for the use of the Kaufman Survey of Early Academic and Language Skills (K-SEALS) were obtained from both the developers of the instrument and the researchers who conducted its Turkish version. Throughout all stages of the research process, the principles of research ethics, scientific integrity, and data reporting standards were strictly followed.

### 2.5 Data collection procedure

The research data were collected in public and private preschool education institutions affiliated with the Turkish Ministry of National Education in Konya after obtaining the required ethical approvals and implementation permissions. The data collection process was conducted between November 2025 and February 2026.

K-SEALS was administered individually to the children by the researcher. Individual administration was used in order to minimize measurement error and to observe children’s performance more accurately. The administrations were conducted in quiet environments free from distracting elements, and each session lasted approximately 20–25 min.

To evaluate test–retest reliability, a convenience subsample of 50 children who had participated in the initial assessment and were available for the second administration was reassessed after a three-week interval. A three-week interval was selected because it is widely recommended in psychometric research as being sufficiently long to minimize recall effects while remaining short enough to reduce the likelihood of true developmental change (DeVellis, 2017). To minimize examiner-related errors, both the initial and the retest administrations were conducted individually by the same researcher in accordance with the standardized administration procedures of the K-SEALS, thereby ensuring consistency throughout the data collection process. No missing data, incomplete administrations, or participant exclusions occurred after data collection; therefore, all 385 participants were included in the analyses.

All data obtained during the research process were anonymized and used solely for scientific purposes. All assessments were administered individually by the same trained researcher following the standardized administration procedures described in the K-SEALS manual.

### 2.6 Data analysis

In the study, confirmatory factor analysis (CFA) was conducted to examine the construct validity of K-SEALS. Since the test items were dichotomous (scored as 0 and 1), the data did not meet the assumption of continuous variables. Accordingly, the Diagonally Weighted Least Squares (DWLS) method was used for parameter estimation. The DWLS method is recommended as an estimation approach for categorical data structures, particularly because it reduces small sample biases and provides more stable estimates under non-parametric distributions.

In the DWLS approach, parameters are estimated using a diagonal weight matrix, and standard errors and model chi-square statistics are calculated using robust mean- and variance-adjusted methods. This approach produces more reliable results in cases where the multivariate normality assumption required by maximum likelihood (ML) estimation is not met. Therefore, the use of robust estimation methods is recommended in the analysis of measurement instruments containing dichotomous or ordinal data (Muthén and Muthén, 2017; Flora and Curran, 2004; Li, 2016).

*Model Fit Evaluation:* Model fit was evaluated using by considering the Comparative Fit Index (CFI), Tucker–Lewis Index (TLI/NNFI), Normed Fit Index (NFI), Goodness of Fit Index (GFI), Root Mean Square Error of Approximation (RMSEA), and Standardized Root Mean Square Residual (SRMR). A multiple-index approach was adopted in evaluating model fit, and interpretations based on a single fit index were avoided. Accordingly:

- CFI and TLI values of 0.90 and above were considered acceptable, and 0.95 and above were considered indicative of good fit,
- RMSEA and SRMR values below 0.08 were considered acceptable, and below 0.05 were considered indicative of good fit (Kline, 2016; Hu and Bentler, 1999).

These threshold values allow for the simultaneous evaluation of both absolute and relative model fit.

*Item-Level Analyses and Model Revision:* In the model improvement process, not only global fit indices but also item-level factor loadings, error variances, and modification indices were evaluated together. In this context, items with low factor loadings (*λ* < 0.30), high error variance, or inconsistency with the theoretical structure were considered for removal during model revision. Final item retention decisions were made by jointly considering statistical evidence and theoretical representativeness.

During the item removal process, not only statistical criteria were considered, but also the theoretical representativeness and content validity of each item were taken into account. Thus, the final model provides a balanced structure in terms of both statistical fit and theoretical integrity.

*Reliability Analyses:* Multiple methods were used to examine the reliability of the scale. First, Cronbach’s alpha coefficient and McDonald’s omega (*ω*) coefficient were calculated to determine internal consistency. Reporting the omega coefficient was preferred, particularly to compensate for the limitations of the alpha coefficient in multidimensional structures.

In addition, test–retest reliability was evaluated using Pearson product–moment correlation coefficients to determine the temporal stability of the measurement instrument.

*Descriptive and Relational Analyses:* Descriptive statistics, including mean, standard deviation, minimum, and maximum values, were calculated to examine the basic statistical characteristics of the K-SEALS subtests and scales. These analyses were conducted to determine the degree of deviation from normality and to identify possible ceiling/floor effects.

Pearson product–moment correlation coefficients were calculated to examine the relationships between subtests and scales. This analysis was used to evaluate whether the dimensions are theoretically related but distinguishable constructs.

*Validity Analyses:* To examine convergent validity, Average Variance Extracted (AVE) and Composite Reliability (CR) values were calculated. An AVE value of 0.50 or above was considered sufficient for convergent validity, while a CR value of 0.70 or above was evaluated as an indicator of sufficient reliability at the factor level (Fornell and Larcker, 1981).

Discriminant validity was evaluated by comparing inter-factor correlations with the square root of AVE values, and the Fornell–Larcker criterion was used. This approach provides strong evidence regarding the discriminability of factors (Hair et al., 2019).

*Developmental Sensitivity Analysis:* To obtain evidence regarding the developmental sensitivity of the scale, the relationships between participants’ chronological ages and the scores obtained from the K-SEALS dimensions were examined using Pearson correlation analysis. This analysis served as an indicator of the instrument’s ability to reflect age-related developmental differences.

*Measurement Invariance Analyses:* Measurement invariance was evaluated across gender groups using multi-group confirmatory factor analysis (MG-CFA). Within this framework, invariance was assessed sequentially at the configural, metric and scalar levels. Developmental sensitivity with respect to age was examined separately through correlation analyses and was not considered part of the measurement invariance procedure.

Differences between models were evaluated by considering the criteria of ΔCFI (≤0.01), ΔRMSEA (≤0.015), and ΔSRMR (≤0.01). These thresholds are based on widely accepted criteria in the international literature for evaluating measurement invariance (Chen, 2007).

*Software and Data Analysis Environment:* All statistical analyses conducted within the scope of the study were performed using IBM SPSS Statistics (version 25) and JASP software packages. Calculations related to factor analysis and structural modeling were carried out through the relevant modules of JASP, and the accuracy of the dataset and the consistency of the model outputs were checked throughout the analysis process.

## 3 Results

In this section, the findings obtained from the analyses conducted regarding the validity and reliability of K-SEALS are presented.

### 3.1 Validity findings

Evidence related to the validity of the Kaufman Survey of Early Academic and Language Skills (K-SEALS) is presented under relevant headings based on the results of confirmatory factor analysis conducted within the scope of construct validity, as well as convergent validity, discriminant validity, and measurement invariance analyses.

#### 3.1.1 Descriptive statistics and relationships among subdomains

The mean, standard deviation, and observed score ranges of the Kaufman Survey of Early Academic and Language Skills are presented in Table 1.

**Table 1:** Descriptive statistics for K-SEALS.

Subtest/Scale	Number of items	Mean (M)	Standard deviation (SD)	Min	Max
Vocabulary (Revised)	30	15.96	7.61	5	30
Numbers–letters–words	40	17.82	8.58	8	40
Articulation skills	20	11.07	5.89	4	20
Expressive language skills	35	15.78	8.20	3	35
Receptive language skills	35	18.00	8.08	6	35
Number skills	19	9.14	4.33	3	19
Letter and word skills	19	7.73	4.42	1	19
Early academic and language skills composite	70	33.78	15.92	13	70

When Table 1 is examined, it is observed that the scores across all subtests and scales are distributed over a wide range and that the variance is at an adequate level. This indicates that the measurement instrument has the capacity to distinguish between different levels of performance.

Due to the ceiling effect observed in the initial items of the Vocabulary subtest, this subtest was evaluated in its revised form. This finding indicates that items with particularly low difficulty levels are insufficient in discriminating high-performing groups and may limit item-level psychometric functioning. The mean score of the revised Vocabulary subtest was found to be 15.96 (SD = 7.61). The mean score of the Numbers, Letters and Words subtest was calculated as 17.82 (SD = 8.58), while the mean score of the Articulation Skills subtest was 11.07 (SD = 5.89).

When the mean values of the subtests and scales are evaluated together, it is observed that there is a relatively balanced distribution between language skills and early academic skills. This suggests that the measurement instrument is capable of assessing different developmental domains simultaneously.

In order to examine the relationships among the three subtests included in the Kaufman Survey of Early Academic and Language Skills, the Language Skills Scales (Expressive Language Skills Scale – Receptive Language Skills Scale), the Early Academic Skills Scales (Number Skills Scale – Letter and Word Skills Scale), and the Early Academic and Language Skills Composite, Pearson product–moment correlation coefficients were calculated. The results are presented in Table 2.

**Table 2:** Correlations among K-SEALS subtests and scales.

Subtest/Scale	*M*	SD	1	2	3	4	5	6	7	8
1. Vocabulary (Revised)	15.96	7.61	—							
2. Numbers–letters–words	17.82	8.58	0.934	—						
3. Articulation skills	11.07	5.89	0.941	0.908	—					
4. Expressive language skills	15.78	8.20	0.958	0.947	0.940	—				
5. Receptive language skills	18.00	8.08	0.961	0.947	0.934	0.942	—			
6. Number skills	9.14	4.33	0.902	0.938	0.861	0.916	0.903	—		
7. Letter and word skills	7.73	4.42	0.893	0.939	0.857	0.913	0.899	0.944	—	
8. Early academic and language skills composite	33.78	15.92	0.981	0.985	0.942	0.978	0.978	0.948	0.945	—

All correlations are significant at p < 0.001.

When Table 2 is examined, it is observed that all relationships among the subdomains are positive and statistically significant. The correlation coefficients were found to range between 0.857 and 0.985. These findings support that the subdimensions of K-SEALS are theoretically related constructs; however, the very high correlation values observed among some subdimensions (particularly *r* ≥ 0.95) raise the question of the extent to which these structures are distinguishable from one another.

In particular, the very high correlation between the Numbers, Letters and Words subtest and the Early Academic and Language Skills Composite (*r* = 0.985) suggests that this composite structure largely encompasses the subdimensions. This situation indicates a pattern that may also be consistent with a hierarchical or higher-order factor structure of the model.

In addition, the high correlations between Vocabulary and Receptive Language (*r* = 0.961) and Expressive Language (*r* = 0.958) indicate that different subcomponents of language skills are strongly related; however, these structures should be supported by confirmatory analyses when modeled as separate factors.

Therefore, rather than interpreting the high correlations among subdimensions in isolation, it is important to evaluate the measurement model as a whole and to consider these findings particularly in conjunction with the results of confirmatory factor analysis.

#### 3.1.2 Construct validity

In order to examine the construct validity of the Kaufman Survey of Early Academic and Language Skills, the eight-factor theoretical model was tested using confirmatory factor analysis. In the analyses, the Diagonally Weighted Least Squares (DWLS) estimation method, which is appropriate for dichotomously scored items, was used.

In the final model, three main subtests (Vocabulary, Numbers, Letters and Words, Articulation Skills), four skill scales (Expressive Language Skills, Receptive Language Skills, Number Skills, Letter and Word Skills), and one composite structure (Early Academic and Language Skills Composite) were evaluated.

The results of the confirmatory factor analysis of the Kaufman Survey of Early Academic and Language Skills are presented in Table 3.

**Table 3:** Confirmatory factor analysis results for K-SEALS.

Factor	Number of items	Factor loading range	Explained variance (*R*^2^)	Mean loading
Vocabulary	30	0.268–0.877	0.072–0.770	0.69
Numbers	20	0.50–0.83	0.25–0.69	0.67
Letters and words	20	0.09–0.87	0.01–0.77	0.61
Articulation skills	10	0.54–0.85	0.29–0.71	0.72
Expressive language skills	2 subtests	0.72–0.91	0.52–0.83	0.84
Receptive language skills	2 subtests	0.70–0.89	0.49–0.79	0.81
Number skills	1 subtest	0.78	0.61	0.78
Letter and word skills	1 subtest	0.81	0.66	0.81

When the confirmatory factor analysis results presented in Table 3 are examined, it is observed that the proposed eight-factor structure of the Kaufman Survey of Early Academic and Language Skills is generally consistent with the data and that the model is supported.

In the Vocabulary subtest, the first 10 items were answered correctly by all participants; therefore, these items did not show variance and were not included in the model. This indicates that these items have low discriminative power and do not contribute to the measurement model. In the revised Vocabulary subtest, standardized factor loadings ranged between 0.268 and 0.877.

In the Numbers, Letters and Words subtest, factor loadings ranged between 0.096 and 0.876; in the Articulation Skills subtest, between 0.540 and 0.845; in the Expressive Language Skills scale, between 0.117 and 0.878; and in the Receptive Language Skills scale, between 0.132 and 0.872. These findings indicate that the majority of the items represent the relevant factors at moderate to high levels. However, the low factor loadings observed in some items suggest that these items may have limited power in representing the measured construct.

In the Number Skills scale, item S23 was removed from the model because it yielded a non-significant factor loading; in the revised model, factor loadings ranged between 0.306 and 0.814. In the Letter and Word Skills scale, item S33 was removed, and in the revised model, factor loadings were calculated to range between 0.473 and 0.858. Following these modifications, the obtained factor loadings indicate that the relevant measurement dimensions achieved a more consistent structure. It should be noted that these item removal decisions apply only to the specific subtest models in which they were identified. Because the composite and skill-scale models were analyzed separately, some items (e.g., S23 and S33) were retained in other measurement models when supported by theoretical considerations and acceptable overall model fit.

In the Early Academic and Language Skills Composite, factor loadings ranged between 0.103 and 0.879. The wide range of factor loadings for the composite structure indicates that the higher-order construct consists of subcomponents contributing at different strengths.

At the subtest level, item factor loadings were generally found to be at moderate to high levels, and all factor loadings were statistically significant (*p* < 0.001). This finding indicates that the items included in the model are statistically related to the measured constructs and that the model overall presents a meaningful structure.

When the factor loadings of the items in the subtests are examined, it is observed that the factors of expressive language skills, receptive language skills, number skills, and letter and word skills are highly represented by their respective subtests.

To provide a clearer overview of the item retention and removal process across the different measurement models, a summary of the item-level decisions is presented in Table 4.

**Table 4:** Item retention and removal decisions across the K-SEALS measurement models.

Item	Measurement model	Decision	Reason
Vocabulary items 1–10	Vocabulary	Removed	No variance
S23	Number skills	Removed	Non-significant factor loading
S33	Letter and word skills	Removed	Non-significant factor loading
S23, S24, S29, S33	Composite model	Retained	Statistically significant in this model and retained to preserve theoretical content representation

When the item retention and removal decisions presented in Table 4 are examined, it is observed that these decisions differed according to the measurement model being evaluated. In the Vocabulary subtest, the first 10 items were excluded because they showed no variance in the present sample. In the Number Skills and Letter and Word Skills models, Items S23 and S33, respectively, were removed because they did not demonstrate adequate psychometric performance. However, these items were retained in other measurement models when supported by the psychometric results of those models together with theoretical considerations. These findings indicate that item retention and removal decisions were made separately for each measurement model according to the psychometric characteristics and theoretical structure of the relevant construct.

In deciding to retain these items in the model, not only statistical criteria but also the theoretical integrity and content representation of the measurement instrument were taken into consideration. Accordingly, it was evaluated that these items had not completely lost their function of representing the measured construct, but contributed relatively less, and they were retained in the analysis in order to preserve the original structure of the scale.

The item parameters obtained from the confirmatory factor analysis for the subtests are presented above. The fit indices of the measurement models are summarized in Table 5 in order to provide a comprehensive evaluation.

**Table 5:** Model fit indices of confirmatory factor analysis for subtests and scales.

Subtest/Scale	CFI	TLI	NFI	GFI	RMSEA	RMSEA (90% CI)	SRMR
Vocabulary	0.987	0.986	0.968	0.970	0.043	0.038–0.048	0.092
Numbers–letters–words	0.967	0.965	0.938	0.945	0.053	0.049–0.056	0.110
Articulation skills	0.967	0.963	0.955	0.956	0.084	0.077–0.091	0.104
Expressive language skills	0.994	0.994	0.974	0.976	0.027	0.021–0.033	0.089
Receptive language skills	0.982	0.981	0.953	0.957	0.040	0.035–0.044	0.093
Number skills	0.973	0.970	0.939	0.951	0.044	0.035–0.052	0.092
Letter and word skills	0.988	0.986	0.966	0.973	0.037	0.028–0.047	0.090
Early academic and language skills composite	0.991	0.991	0.967	0.969	0.030	0.027–0.033	0.089

CFI = Comparative Fit Index; TLI = Tucker–Lewis Index; NFI = Normed Fit Index; GFI = Goodness-of-Fit Index; RMSEA = Root Mean Square Error of Approximation; CI = Confidence Interval; SRMR = Standardized Root Mean Square Residual.

When Table 5 is examined, it is observed that the CFI values range between 0.967 and 0.994, the TLI/NNFI values range between 0.963 and 0.994, and the NFI values range between 0.938 and 0.974. These findings indicate that the relative fit of the model is high for all subtests and scales and that the data are largely consistent with the proposed factor structure.

It was determined that the RMSEA values range between 0.027 and 0.084. It is observed that the majority of the RMSEA values are below 0.05 or fall within the 0.05–0.08 range, indicating that the model generally demonstrates good and acceptable levels of absolute fit. However, it is seen that the RMSEA value for the Articulation Skills subtest (0.084) is at the threshold of acceptable fit.

The SRMR values were found to range between 0.089 and 0.110. Although SRMR values in some subtests exceed the recommended threshold of 0.08, this was not interpreted as a level of misfit that would weaken the overall model fit when evaluated together with the high values of other fit indices. It is recommended that model fit should be evaluated based on multiple fit indices rather than relying on a single index. It should be noted that residual-based fit indices such as SRMR and RMSEA may be more sensitive in multidimensional models estimated with categorical (dichotomous) data, which may partially explain the slightly elevated values observed in some subtests.

In this context, the high values of incremental fit indices such as CFI and TLI support that the model is generally consistent with the data. Overall, the obtained fit indices suggest that the proposed factor structure of K-SEALS at the subtest and scale levels demonstrates acceptable and largely good fit with the data. Given the multidimensional structure of the model and the use of DWLS estimation with dichotomous data, slightly elevated SRMR values may be expected and should be interpreted with caution in conjunction with other fit indices.

#### 3.1.3 Convergent validity findings (AVE and CR)

In order to determine the extent to which the factors in the measurement model represent their respective constructs and whether the items measuring the same construct are consistent with each other, convergent validity analysis was conducted. Convergent validity is considered one of the fundamental components of construct validity, as it reveals the extent to which the items forming a factor explain a common underlying structure (Fornell and Larcker, 1981).

To evaluate the convergent validity of K-SEALS, Average Variance Extracted (AVE) and Composite Reliability (CR) values were calculated for each factor. While the AVE value represents the average variance explained by the items of a factor, the CR value provides an indicator of the composite reliability of the respective factor. In the literature, an AVE value of 0.50 or above indicates that the factor is adequately represented by its items, whereas a CR value of 0.70 or above indicates sufficient reliability at the factor level (Fornell and Larcker, 1981; Hair et al., 2019).

In this context, the joint evaluation of AVE and CR values provides a holistic understanding of both the internal consistency and explanatory power of the factors included in the measurement model. The convergent validity findings of K-SEALS are presented in Table 6.

**Table 6:** Convergent validity results for K-SEALS (AVE and CR).

Subtest/Scale	AVE	CR
Vocabulary	0.52	0.91
Numbers–letters–words	0.49	0.90
Articulation skills	0.55	0.92
Expressive language skills	0.58	0.93
Receptive language skills	0.56	0.92
Number skills	0.47	0.86
Letter and word skills	0.51	0.88
Early academic and language skills composite	0.63	0.95

AVE = Average Variance Extracted; CR = Composite Reliability.

When Table 6 is examined, it is observed that CR values for all subtests and scales range between 0.86 and 0.95. This finding indicates that all factors have a high level of internal consistency and composite reliability. AVE values were found to range between 0.47 and 0.63.

It is observed that AVE values are 0.50 or above for Vocabulary, Articulation Skills, Expressive Language Skills, Receptive Language Skills, Letter and Word Skills, and the Early Academic and Language Skills Composite. It can be stated that convergent validity criteria are met for these factors.

However, it was determined that AVE values for the Numbers, Letters and Words (AVE = 0.49) and Number Skills (AVE = 0.47) subdimensions are below the 0.50 threshold value. This indicates that the level at which the items explain the common variance within these factors is limited.

Nevertheless, the CR values calculated for these factors are above the 0.70 threshold and at a high level, indicating that the internal consistency of these factors is strong and that the measured construct is reliably represented. In the literature, it is stated that when the AVE value is below 0.50, convergent validity should not be completely rejected if the CR value is high, and such cases may be considered acceptable (Fornell and Larcker, 1981).

Accordingly, when considered together, the findings suggest that the K-SEALS subtests and scales demonstrate generally adequate convergent validity and high composite reliability.

#### 3.1.4 Discriminant validity findings (Fornell–Larcker)

The Fornell–Larcker criterion was used to evaluate the discriminant validity of K-SEALS. Discriminant validity is an indicator of construct validity that demonstrates the extent to which the factors in the measurement model are distinguishable from one another and plays a critical role in determining whether each factor represents a unique construct (Fornell and Larcker, 1981).

Establishing discriminant validity is particularly important in cases where high correlations are observed among factors, as it allows for the evaluation of whether the multidimensional structure of the measurement model is preserved. Within this scope, the square root of the AVE values calculated for each factor was compared with the inter-factor correlation coefficients.

According to the Fornell–Larcker criterion, in order to establish discriminant validity, the square root of the AVE value for each factor must be greater than the correlation coefficients of that factor with other factors (Fornell and Larcker, 1981; Hair et al., 2019). This approach enables the evaluation of whether the variance explained by each factor through its own items is greater than the variance shared with other factors, and thus reveals whether the level of discrimination among factors is sufficient.

The discriminant validity findings of K-SEALS are presented in Table 7.

**Table 7:** Fornell–Larcker discriminant validity matrix of K-SEALS.

Subtest/Scale	1	2	3	4	5	6	7	8
1. Vocabulary	**0.72**							
2. Numbers–letters–words	0.63	**0.70**						
3. Articulation skills	0.61	0.59	**0.74**					
4. Expressive language skills	0.67	0.64	0.60	**0.76**				
5. Receptive language skills	0.66	0.63	0.58	0.69	**0.75**			
6. Number skills	0.58	0.66	0.55	0.61	0.60	**0.69**		
7. Letter and word skills	0.57	0.67	0.54	0.60	0.59	0.71	**0.71**	
8. Early academic and language skills composite	0.79	0.81	0.72	0.78	0.77	0.70	0.69	**0.79**

Bold values on the diagonal represent the square root of the Average Variance Extracted (AVE), used to assess discriminant validity according to the Fornell–Larcker criterion.

The Fornell–Larcker discriminant validity matrix for K-SEALS is presented in Table 7. The diagonal values represent the square root of the AVE for each factor, while the off-diagonal values represent the inter-factor correlation coefficients.

When Table 7 is examined, the results showed for the majority of the factors, the square root of the AVE values is higher than the correlation coefficients between the relevant factor and the other factors. These findings suggest that discriminant validity was largely supported. However, for the Early Academic and Language Skills Composite factor, the correlation coefficients with some factors are very close to, or in some cases exceed, the square root of the AVE of the corresponding factor. This finding indicates substantial conceptual overlap among some theoretically related constructs, particularly those involving the composite factor. Nevertheless, this pattern may be expected because early academic and language skills are developmentally highly interrelated constructs. Therefore, the findings provide substantial, although not unequivocal, evidence for discriminant validity. In addition, the composite scoring structure of the instrument may have contributed to the high inter-factor correlations, suggesting the presence of a higher-order latent structure that could be examined in future studies using higher-order or bifactor models. Overall, the discriminant validity findings of K-SEALS can be considered largely acceptable. Nevertheless, the high correlations observed among several theoretically related factors indicate that the constructs are not entirely distinct and should therefore be interpreted with appropriate caution.

#### 3.1.5 Measurement invariance

When the literature on measurement invariance is examined, the results showed there is no complete consensus regarding the criteria used. In the review study conducted by Putnick and Bornstein (2016), it was reported that different decision criteria are used together in measurement invariance analyses. This situation necessitates adopting a multiple-index approach rather than relying on a single statistical criterion when evaluating measurement invariance. In the present study, measurement invariance analyses were conducted only across gender groups. Age-related evidence was evaluated separately through developmental sensitivity analyses.

Accordingly, considering the limitations of the Δχ^2^ statistic which has high sensitivity in large samples and may render even small differences statistically significant (Chen, 2007; Cheung and Rensvold, 2002) ΔCFI, ΔRMSEA, and ΔSRMR values were jointly taken into account in the evaluation of measurement invariance.

In the literature, threshold values of ≤ 0.01 for ΔCFI (Cheung and Rensvold, 2002) and in some cases ≤ 0.02 (Rutkowski and Svetina, 2014) are recommended; for ΔRMSEA and ΔSRMR, threshold values of ≤ 0.015 are suggested (Chen, 2007). In this study, a more conservative approach was adopted, and the criteria of ΔCFI ≤ 0.01–0.02, ΔRMSEA ≤ 0.015, and ΔSRMR ≤ 0.015 were used as the basis.

The CFI, ΔRMSEA, and ΔSRMR values obtained from the measurement invariance analyses for subtests and scales are presented in Table 8.

**Table 8:** Measurement invariance results for subtests and scales.

Subtest/Scale	ΔCFI	ΔRMSEA	ΔSRMR
Vocabulary	0.016	0.006	0.008
Numbers–letters–words	0.012	0.006	0.014
Articulation skills	0.018	0.029	0.014
Expressive language skills	0.006	0.004	0.007
Receptive language skills	0.004	0.008	0.023
Number skills	0.003	0.009	0.005
Letter and word skills	0.002	0.009	0.006
Early academic and language skills composite	0.003	0.003	0.004

ΔCFI, ΔRMSEA, and ΔSRMR represent the changes between successive measurement invariance models (configural, metric, and scalar).

When Table 8 is examined, it is observed that for the majority of subtests and scales, ΔCFI, ΔRMSEA, and ΔSRMR values remain within the recommended threshold values. This finding suggests that K-SEALS functions in a largely similar manner across gender groups and provides evidence supporting measurement invariance. These findings suggest that configural and metric invariance across gender groups were largely supported, whereas scalar invariance should be interpreted with caution for certain subtests. However, the results showed the ΔRMSEA value for the Articulation Skills subtest (0.029) exceeds the recommended threshold value, and that the ΔCFI values for the Vocabulary (0.016) and Articulation Skills (0.018) subtests are at the borderline level. These findings do not indicate that measurement invariance is not established in some subtests; rather, they suggest that the results should be interpreted more cautiously.

In particular, these differences observed in language-based subtests may reflect the developmental and linguistic characteristics of the measured skills and therefore should be interpreted with caution. Overall, the findings indicate that K-SEALS largely demonstrates measurement invariance across gender groups at the level of subtests and scales, although limited differences were observed in some language-related subdimensions. Developmental sensitivity with respect to age was evaluated separately through correlation analyses and is presented in the following section.

### 3.2 Reliability findings

When Table 9 is examined, the results showed test–retest correlations range between 0.980 and 0.996, McDonald’s omega coefficients range between 0.862 and 0.981, and Cronbach’s alpha coefficients range between 0.828 and 0.956. The obtained findings indicate that K-SEALS demonstrates high internal consistency at the level of all subtests and scales and that the measurements are highly stable over time.

**Table 9:** Reliability results for K-SEALS subtests and scales.

Subtest/Scale	Test–retest (*r*)	McDonald’s *ω*	Cronbach’s *α*
Vocabulary	0.992	0.943	0.917
Numbers–letters–words	0.989	0.948	0.913
Articulation skills	0.993	0.954	0.906
Expressive language skills	0.990	0.945	0.922
Receptive language skills	0.995	0.936	0.909
Number skills	0.986	0.862	0.828
Letter and word skills	0.980	0.893	0.857
Early academic and language skills composite	0.996	0.981	0.956

Test–retest correlations represent Pearson correlation coefficients between two measurement occasions.

In particular, the high McDonald’s omega coefficients support that the factors are reliably measured despite the multidimensional structure of the scale. Cronbach’s alpha coefficients above 0.80 indicate that the internal consistency of the measurement instrument is sufficient and at a high level.

The very high test–retest correlations (0.980–0.996) indicate that the measurements are consistent over time and that the scale produces stable measurements. Therefore, these values should be interpreted not only as indicators of temporal stability but also in relation to the relatively short retest interval and controlled administration conditions. However, such high correlation values may be related to the lack of change in the measured construct over a short time interval and the similarity of the administration conditions. These values may also reflect the short retest interval and highly standardized administration conditions rather than solely the intrinsic temporal stability of the construct.

Overall, the findings provide further evidence supporting the reliability of K-SEALS in terms of internal consistency and temporal stability. These very high test–retest coefficients may also be influenced by the relatively short time interval between administrations and the highly standardized testing conditions, rather than solely reflecting the inherent temporal stability of the measured constructs.

When Table 10 is examined, the findings indicated the relationships between age and subdomain scores are at a low level, with correlation coefficients ranging between 0.006 and 0.040. All obtained correlations are statistically non-significant (*p* > 0.05). These findings indicate that, in the examined sample, K-SEALS subtest and scale scores do not show a significant relationship with chronological age.

**Table 10:** Correlations between age and K-SEALS subtests and scales.

Subtest/Scale	*r*	*p*
Vocabulary (Revised)	0.019	0.711
Numbers–letters–words	0.034	0.503
Articulation skills	0.006	0.902
Expressive language skills	0.040	0.433
Receptive language skills	0.014	0.791
Number skills	0.019	0.716
Letter and word skills	0.038	0.456
Early academic and language skills composite	0.028	0.590

Although it is developmentally expected that academic and language skills increase with age during early childhood, the absence of significant relationships in this study may be explained by several possible factors. This pattern may be explained by restricted variance in the age variable, as the sample was largely concentrated within a narrow developmental range, which may have limited the ability to detect age-related differences. First, the fact that the sample is largely concentrated in the 60–72 month age range may have limited the variance in the age variable, thereby resulting in low correlations. In addition, the ceiling effect observed in some subtests (particularly in the Vocabulary subtest) and the generally high performance levels may have limited the discriminative power of the measurement instrument among high-performing groups.

This finding should not be interpreted as indicating a complete absence of developmental sensitivity; rather, it suggests that developmental sensitivity may have been observed at a limited level within the current sample and data distribution.

## 4 Discussion

In this study, the psychometric properties of the Kaufman Survey of Early Academic and Language Skills (K-SEALS) were re-evaluated in a sample of Turkish children aged 48–72 months within the framework of contemporary analytical techniques and a multidimensional approach. The findings indicate that the theoretically proposed structure of the scale is generally preserved; however, they also suggests patterns that require careful interpretation in terms of inter-factor relationships, item-level functioning, and contextual sensitivity.

The results of the confirmatory factor analysis suggest that the eight-factor structure of K-SEALS is generally confirmed in the Turkish sample. Nevertheless, the low factor loadings observed in some items and the removal of certain items from the model indicate that not all components of the measurement instrument possess the same level of representational power. This finding is consistent with the literature emphasizing the heterogeneous and multi-layered nature of early academic and language skills (Duncan et al., 2007; Snow and Matthews, 2016). It also aligns with the psychometric perspective supporting the necessity of re-testing measurement instruments across different samples. Accordingly, the low factor loadings observed in some items should not be interpreted as evidence against the overall validity of the instrument. Rather, they indicate that certain items contribute less strongly to their intended constructs within the present sample. This finding highlights the importance of evaluating item-level performance alongside overall model fit when interpreting multidimensional measurement instruments.

When model fit indices are examined, it is observed that an overall good and acceptable level of fit has been achieved. In particular, the high CFI and TLI values strongly support the relative fit of the model. However, the borderline RMSEA and SRMR values observed in some subtests suggest that the model may exhibit a more complex or heterogeneous structure in certain dimensions. This is consistent with studies indicating that some fit indices may be more sensitive in multidimensional analyses conducted with dichotomous data (Hu and Bentler, 1999; Kline, 2016).

The examination of relationships among the K-SEALS dimensions revealed that correlation coefficients were quite high (*r* ≈ 0.85–0.98). This finding suggests that early academic and language skills function not as independent constructs but as strongly interactive and integrated components within a holistic developmental system. This result is consistent with sociocultural theory (Vygotsky, 1978), the executive functions framework (Diamond, 2013), and interactional developmental models (Sameroff, 2009). However, such high correlations also raise the possibility of content overlap or structural convergence among subdimensions. This pattern is partially reflected in the discriminant validity findings. Fornell–Larcker analyses indicate that, although factors are largely distinguishable, there is limited discriminant validity particularly between the composite structure and some subdimensions. This may suggest that cognitive, linguistic, and academic skills in early childhood are not yet fully differentiated. At the same time, this pattern may support the theoretical presence of a higher-order structure within the measurement model. From a psychometric perspective, these findings suggest that the observed multidimensional structure may also be represented by a higher-order latent construct underlying the related subdimensions. Although the present study was designed to evaluate the originally proposed multidimensional structure of K-SEALS, future studies may compare the current model with higher-order and bifactor models to determine whether these alternative representations provide a better fit to the data. Such analyses may contribute to a more comprehensive understanding of the hierarchical organization of early academic and language skills.

When convergent validity findings are examined, the results showed AVE values are acceptable for most subdimensions, while some remain at borderline levels. However, the high CR values obtained for all factors indicate that the constructs are reliably represented. This finding is consistent with the literature emphasizing that AVE and CR should be evaluated together, especially in multidimensional structures (Fornell and Larcker, 1981). Overall, the findings suggest that the scale provides adequate convergent validity, although the explained variance may be limited in some subdimensions.

Measurement invariance analyses indicate that K-SEALS measures largely similar constructs across gender groups. However, the limited deviations observed in some subtests may be related to the context-sensitive nature of language-based skills. This finding suggests that measurement instruments may not present completely invariant structures across groups, but rather exhibit a certain level of contextual variation.

Reliability findings indicate that the scale demonstrates strong internal consistency and temporal stability. In particular, the high McDonald’s omega coefficients support that factors are reliably measured despite the multidimensional structure. The very high test–retest coefficients suggest that the measurements remained highly consistent over the 3-week interval. However, these coefficients may also reflect the relatively short retest interval and the highly standardized administration conditions.

One of the noteworthy findings of the study is the absence of a significant relationship between age and subdomain scores. Although these skills are developmentally expected to increase with age, the lack of significant relationships may be explained by the narrow age range of the sample and the limited discriminative power of the measurement instrument at higher performance levels. This finding is also consistent with developmental approaches emphasizing that early childhood development is shaped not only by chronological age but also by environmental inputs, learning experiences, and linguistic interactions (Bronfenbrenner and Morris, 2006; Pace et al., 2017).

The findings obtained are generally consistent with previous psychometric evidence regarding the Turkish adaptation of K-SEALS; however, the present study provides a more advanced and detailed examination of these findings. In the adaptation study conducted by Uyanık and Kandır (2014), it was reported that the theoretical structure of the scale was largely preserved and that internal consistency coefficients were at acceptable levels. In the present study, the more comprehensive examination of both confirmatory factor analysis results and internal consistency coefficients provides stronger and more up-to-date psychometric evidence regarding the functioning of K-SEALS in the Turkish sample. In particular, the high Cronbach’s alpha and McDonald’s omega coefficients indicate that the scale produces not only acceptable but also highly consistent measurements.

However, the high correlations observed among subdimensions (*r* ≈ 0.85–0.98) suggest a more holistic structural pattern compared to previous adaptation findings. While the original K-SEALS technical report indicated more moderate relationships among subdimensions (Kaufman and Kaufman, 1993), the high correlations obtained in this study suggest that early academic and language skills may emerge as more strongly overlapping constructs in the Turkish sample. This may be explained by the not-yet fully differentiated nature of cognitive, linguistic, and academic skills in early childhood, as well as the narrow age range of the sample and the composite scoring structure of the instrument.

This difference appears to be influenced not only by developmental characteristics but also by linguistic and cultural context. Cross-cultural measurement literature emphasizes that measurement instruments may differ across languages not only at the level of translation but also in terms of structural features and usage contexts of the language (Hambleton, 2005; Van de Vijver and Tanzer, 2004). The agglutinative structure of Turkish may represent one of several possible explanations for the more holistic performance pattern observed, particularly in expressive and receptive language skills. Therefore, the high correlations observed among the K-SEALS dimensions may reflect not only characteristics of the measurement model but also aspects of the linguistic structure of Turkish. However, this interpretation should be considered tentative because these mechanisms were not directly examined in the present study.

This interpretation is also consistent with findings reported in the K-SEALS technical manual. It has been noted that the same measurement instrument may produce different performance patterns across linguistic groups and that performance in subtests such as vocabulary may be related more to linguistic familiarity than to cognitive ability (Kaufman and Kaufman, 1993). In this context, the low variance, ceiling effects, and relatively low factor loadings observed in some items in the present study indicate that the measurement instrument may function differently across specific samples. Particularly in early childhood, some items may become too easy, thereby limiting discriminative power among high-performing groups. In this respect, the study not only confirms the structural validity of the scale but also provides a more detailed evaluation of item-level functioning. Therefore, although these items exhibited relatively low factor loadings, their evaluation contributed to identifying aspects of the instrument that may require further refinement in future applications. At the same time, the overall factorial structure remained stable, supporting the continued use of the scale in Turkish preschool children.

The findings also suggest that early academic and language skills may be associated not only with cognitive development but also with environmental and linguistic factors. Language development is known to be closely related to family environment, quality of interaction, and diversity of language input (Pace et al., 2017). Therefore, the holistic structure observed particularly in language-based subdimensions suggests that these skills are shaped not only by individual capacity but also by learning experiences and sociocultural context. Studies conducted in different cultural contexts similarly demonstrate that language and academic performance are associated not only with developmental level but also with linguistic background and environmental experiences.

Another notable finding is the lack of significant relationships between age and subdomain scores. Although this initially appears to contradict developmental expectations, it may be explained by the limited age variance within the sample, the relatively homogeneous characteristics of the study sample, and the ceiling effects observed in some subtests, which may have limited the discriminative power of the instrument at higher performance levels. Therefore, this finding does not indicate a complete absence of developmental sensitivity but rather suggests that it may not have been sufficiently captured within the current sample structure. This interpretation is consistent with developmental approaches emphasizing that early childhood development should be understood not only in terms of chronological age but also in relation to environmental and experiential factors (Bronfenbrenner and Morris, 2006).

Overall, the findings suggest that the Turkish version of K-SEALS demonstrates satisfactory psychometric properties in children aged 48–72 months. The present study provides further psychometric evidence supporting its construct validity, reliability, and measurement equivalence while extending the available evidence through the application of contemporary psychometric methods. In addition, the findings highlight the potential influence of linguistic, cultural, and developmental contexts on the assessment of early academic and language skills, although these influences should be investigated directly in future research.

## 5 Conclusion

In this study, the psychometric properties of the Kaufman Survey of Early Academic and Language Skills (K-SEALS) were re-evaluated in a sample of Turkish children aged 48–72 months using comprehensive and multidimensional analytical approaches. The findings indicate that the theoretically proposed multidimensional structure of the scale is generally supported in the Turkish sample and that the measurement model provides further psychometric evidence regarding its validity and reliability.

The results obtained from confirmatory factor analysis, convergent and discriminant validity, and measurement invariance analyses suggest that K-SEALS is a structurally consistent measurement instrument that is largely comparable across different groups. Reliability findings further support that the scale provides high internal consistency and temporal stability.

However, the findings offer not only evidence of psychometric adequacy but also important insights into the nature of the constructs being measured. The high level of relationships observed among subdimensions indicates that early academic and language skills should be considered as part of a holistic and interactive developmental system rather than as independent constructs. The partially limited discriminant validity findings further support this holistic structure.

On the other hand, the low variance and ceiling effects observed in some items reveal that language-based items are sensitive to cultural and linguistic context. This suggests that measurement instruments may differ across languages not only structurally but also in terms of item functioning and inter-factor relationships. The agglutinative structure of Turkish and differences in learning experiences may represent possible explanations for this pattern. However, these mechanisms were not directly examined in the present study and therefore should be interpreted with caution.

Additionally, the absence of a significant relationship between age and subdomain scores indicates that early childhood development cannot be explained solely by chronological age; environmental inputs, learning experiences, and linguistic interactions may also contribute to developmental differences.

Overall, the findings suggest that the Turkish version of K-SEALS demonstrates satisfactory psychometric properties in children aged 48–72 months. The present study provides further psychometric evidence supporting its construct validity, reliability, and measurement equivalence while extending the available evidence through the application of contemporary psychometric approaches.

In this respect, the study does not merely provide an adaptation validation; it also offers an original contribution by extending the psychometric evidence available for the Turkish version of K-SEALS and highlighting the importance of re-evaluating measurement instruments across different contexts.

## 6 Limitations

First, the concentration of the sample within a relatively narrow age range (particularly 5–6 years) and the relatively homogeneous characteristics of the study sample may have limited developmental variance and contributed to higher correlations among variables. This may also explain the absence of statistically significant relationships between age and subtest scores.

Second, the selection of participants from similar educational environments (schooling effect) may have reduced variability in learning experiences, potentially leading to more homogeneous performance patterns. Future studies should include more diverse samples in terms of socioeconomic background, parental educational level, and educational settings to enhance the generalizability of the findings and to further evaluate the developmental sensitivity of the instrument across more heterogeneous populations.

Third, the presence of ceiling effects and low variance in some items may have limited item discrimination, particularly for language-based items. This finding highlights the importance of examining item-level functioning when adapting measurement instruments across different linguistic contexts.

In addition, the cross-sectional design of the study does not allow for direct examination of developmental change over time. Longitudinal studies are recommended to better understand the developmental trajectories of early academic and language skills.

Finally, measurement invariance analyses in this study were limited to specific demographic variables. Future research should examine invariance across more diverse cultural, linguistic, and socioeconomic groups to provide more comprehensive evidence for cross-cultural applicability.

## 7 Implications and recommendations

Based on the findings obtained from the study, the following recommendations can be presented for both research and practice:

### 7.1 Research-oriented recommendations

- It is recommended that K-SEALS be re-examined with larger samples covering different age groups and using longitudinal designs.
- It may be beneficial to evaluate the scale across different socioeconomic, cultural, and linguistic contexts through measurement invariance analyses.
- It may be considered to examine the item-level functioning of the scale in greater detail by using Item Response Theory (IRT) and Differential Item Functioning (DIF) analyses.
- Considering the ceiling effects observed particularly in language-based items, it may be appropriate to conduct new item development studies aimed at increasing item discrimination.

### 7.2 Practice- and education-oriented recommendations

- In early childhood assessment processes, it may be beneficial to consider children’s performance through a multidimensional and holistic approach rather than focusing on a single skill domain.
- It may be recommended that teachers take into account the interaction among skills when interpreting assessment results.
- It may be appropriate to structure early childhood education programs in a way that holistically supports language and early academic skills.
- It may be considered to develop educational policies that support the use of scientifically grounded measurement instruments in early childhood.
- It may be beneficial to increase in-service training programs aimed at improving teachers’ competencies in assessment and evaluation.

## Data Availability

The raw data supporting the conclusions of this article will be made available by the authors, without undue reservation.
